# *Aspergillus fumigatus* High Osmolarity Glycerol Mitogen Activated Protein Kinases SakA and MpkC Physically Interact During Osmotic and Cell Wall Stresses

**DOI:** 10.3389/fmicb.2019.00918

**Published:** 2019-05-07

**Authors:** Adriana Oliveira Manfiolli, Eliciane Cevolani Mattos, Leandro José de Assis, Lilian Pereira Silva, Mevlüt Ulaş, Neil Andrew Brown, Rafael Silva-Rocha, Özgür Bayram, Gustavo H. Goldman

**Affiliations:** ^1^Faculdade de Ciências Farmacêuticas de Ribeirão Preto, Universidade de São Paulo, Ribeirão Preto, Brazil; ^2^Department of Biology, Maynooth University, Maynooth, Ireland; ^3^Department of Biology and Biochemistry, University of Bath, Bath, United Kingdom; ^4^Faculdade de Medicina de Ribeirão Preto, Universidade de São Paulo, Ribeirão Preto, Brazil

**Keywords:** *Aspergillus fumigatus*, mitogen activate protein kinase, HOG, MPKC, SakA

## Abstract

*Aspergillus* *fumigatus*, a saprophytic filamentous fungus, is a serious opportunistic pathogen of mammals and it is the primary causal agent of invasive aspergillosis (IA). Mitogen activated protein Kinases (MAPKs) are important components involved in diverse cellular processes in eukaryotes. *A. fumigatus* MpkC and SakA, the homologs of the *Saccharomyces cerevisiae* Hog1 are important to adaptations to oxidative and osmotic stresses, heat shock, cell wall damage, macrophage recognition, and full virulence. We performed protein pull-down experiments aiming to identify interaction partners of SakA and MpkC by mass spectrometry analysis. In presence of osmotic stress with sorbitol, 118, and 213 proteins were detected as possible protein interactors of SakA and MpkC, respectively. Under cell wall stress caused by congo red, 420 and 299 proteins were detected interacting with SakA and MpkC, respectively. Interestingly, a group of 78 and 256 proteins were common to both interactome analysis. Co-immunoprecipitation (Co-IP) experiments showed that SakA::GFP is physically associated with MpkC:3xHA upon osmotic and cell wall stresses. We also validated the association between SakA:GFP and the cell wall integrity MAPK MpkA:3xHA and the phosphatase PtcB:3xHA, under cell wall stress. We further characterized *A. fumigatus* PakA, the homolog of the *S. cerevisiae* sexual developmental serine/threonine kinase Ste20, as a component of the SakA/MpkC MAPK pathway. The *ΔpakA* strain is more sensitive to cell wall damaging agents as congo red, calcofluor white, and caspofungin. Together, our data supporting the hypothesis that SakA and MpkC are part of an osmotic and general signal pathways involved in regulation of the response to the cell wall damage, oxidative stress, drug resistance, and establishment of infection. This manuscript describes an important biological resource to understand SakA and MpkC protein interactions. Further investigation of the biological roles played by these protein interactors will provide more opportunities to understand and combat IA.

## Introduction

Protein-protein interactions (PPI) are essential for the accomplishment of many biological functions (Bork et al., 2004). Interactome analyses can increase the understanding of the biological interactions and cellular processes within an organism. Recent high-throughput studies have obtained a great amount of PPI data, including model organisms such as *Saccharomyces cerevisiae* (Lam et al., 2015) and *Aspergillus nidulans* (Bayram et al., 2012; Jaimes-Arroyo et al., 2015), as well as plant pathogens *Fusarium* *graminearum* (Zhao et al., 2009), *Magnaporthe grisea* (He et al., 2008), and *Phomopsis longicolla* (Li et al., 2018). Bacteria, plants (Nishiyama et al., 2013), and fungi (Aguirre et al., 2006) use conserved phosphorelay systems to sense different types of environmental stresses. Mitogen activated protein Kinases (MAPKs) are important components involved in diverse cellular processes in eukaryotes (Pearson et al., 2001). Filamentous fungi contain homologous MAPKs which mediate specific cell signaling events and coordinated the appropriate biological response. MAPK pathways control the response to multiple stresses including, oxidative, osmotic, heat shock, reactive oxygen species, nutrient limitation, and high concentrations of heavy metals (Rispail et al., 2009; Hamel et al., 2012; Turra et al., 2014).

In *S. cerevisiae*, MAPK Hog1p is involved in many aspects of the osmotic stress response, such as ion transport across cell membranes, cell cycle progression, and regulation of transcription and translation processes (Martínez-Montañés et al., 2010; de Nadal and Posas, 2015). In *A. nidulans*, a conserved phosphorelay cascade activates the MAPK SakA/HogA (Vargas-Perez et al., 2007) in response to oxidative, osmotic, and nutrient starvation stresses (Kawasaki et al., 2002; Lara-Rojas et al., 2011). SakA is a *S. cerevisiae* Hog1p homolog and in association with the AtfA transcription factor was shown to be involved in osmotic stress response (Hagiwara et al., 2009). In addition, SakA phosphorylation is required for asexual and sexual development (Lara-Rojas et al., 2011). During oxidative stress, SakA interacts with AtfA, which then activates the catalase genes *catA* and *catB* (Lara-Rojas et al., 2011). The *sakA* and *atfA* deletion mutants are sensitive to oxidative stress. In other filamentous fungi the SakA/Hog1p orthologs have been shown to have roles in osmotic and oxidative stress responses, while also being involved the regulation of development, and/or virulence (Eaton et al., 2008; Heller et al., 2012; Lamb et al., 2012; Van Nguyen et al., 2013; Nimmanee et al., 2015).

*Aspergillus fumigatus* is a saprophytic filamentous fungus and a deadly opportunistic pathogen of mammals (Greenberger, 2002; Dagenais and Keller, 2009). It is the primary causal agent of Invasive Aspergillosis (IA), one of the most common life threatening fungal diseases in neutropenic patients and has been shown to have mortality rates that can reach 90% (Brakhage, 2005; Brown et al., 2012a,b; Lackner and Lass-Flörl, 2013). It has been reported that several phenotypes influence the final outcome of the IA establishment showing that aspergillosis is a multifactorial disease (Tekaia and Latgé, 2005; Hartmann et al., 2011; Sugui et al., 2014). The signaling pathways that regulate these factors involved in virulence are essentials for *A. fumigatus* survival within the human host (Brown and Goldman, 2016). As many filamentous fungi, *A. fumigatus* has also four different MAPKs named as MpkA, MpkB, MpkC, and SakA. The MpkA function is mainly related to cell wall integrity (CWI, Valiante et al., 2015a). The MpkB, which is homologous to yeast Fus3, has not been yet characterized (Elion et al., 1990). In *A. fumigatus*, MpkC and SakA are homologs of the *S. cerevisiae* Hog1 and are the major regulators of the osmotic stress response (Rispail et al., 2009). MpkC and SakA also play a role in carbon source utilization and caspofungin adaptation, respectively (Reyes et al., 2006; Altwasser et al., 2015; Valiante et al., 2015b).

Our group recently showed that MpkC and SakA are important to adaptations to oxidative and osmotic stresses, heat shock, and cell wall damage (de Oliveira Bruder Nascimento et al., 2016). The double mutant *ΔmpkC ΔsakA* demonstrated increased sensitivity to the above mentioned stresses when compared to the *ΔsakA* and *ΔmpkC* single mutants. In addition, this interaction was crucial for macrophage recognition and full virulence. In most of stress conditions tested, the phenotypes of *ΔsakA* were intensified by the *ΔmpkC* mutation, while *ΔmpkC* phenotypes were moderate (de Oliveira Bruder Nascimento et al., 2016). Accordingly, we proposed that SakA and MpkC are interactive and that MpkC could be a modulator of SakA during HOG and CWI pathways (de Oliveira Bruder Nascimento et al., 2016).

No information is available concerning the SakA and MpkC protein targets during osmotic and cell wall stresses, and very little is known about the mechanisms by which SakA and MpkC control these stress responses. Our aim was to identify SakA and MpkC targets that could mediate their functions. We demonstrate that SakA and MpkC show functional and physical interactions and that these MAPKs of the HOG pathway play important roles in the CWI pathway. We report that the identified PakA kinase, similar to Ste20, a HOG pathway protein in *S. cerevisiae*, and the MpkA central regulator of CWI pathway, associate with SakA/MpkC MAPK pathway. Finally, we show that during cell wall stress, SakA associates with PtcB, a HOG response phosphatase, involved in regulation of MpkA and SakA phosphorylation. This manuscript provides an important resource to understand SakA and MpkC protein interactions in response to osmotic stress and cell wall damage. Further investigation of the biological roles played by these protein interactors will provide more opportunities to investigate and combat IA.

## Results

### Identification of Proteins That Interact With SakA and MpkC During Osmotic and Cell Wall Stresses

In *A. fumigatus* SakA and its paralog MpkC, are involved in osmotic stress, nitrogen and carbon starvation, caspofungin tolerance and are important to adaptations to oxidative stress, heat shock, and cell wall damage (Reyes et al., 2006; Rispail et al., 2009; Altwasser et al., 2015; Valiante et al., 2015a; de Oliveira Bruder Nascimento et al., 2016). In an attempt to further explore the regulatory processes and diverse functions of SakA and MpkC, we sought to identify their interacting protein partners. As previously shown the SakA:GFP and MpkC:GFP strains are functional (de Oliveira Bruder Nascimento et al., 2016). We performed protein pull-down experiments aiming to identify interaction partners of SakA and MpkC by mass spectrometry assays. Protein extracts were prepared from wild-type, SakA:GFP and MpkC:GFP cultures grown for 24 h and further exposed to sorbitol 1.0 M (10, 30, and 60 min) or CR 300 μg/ml (5, 15, 30, and 60 min). We compared proteins that immunoprecipitated with the SakA:GFP or MpkC:GFP and wild-type negative control, removing proteins that precipitated with the latter because they are potential artifacts (Supplementary Tables S1–S8). The full group of proteins that co-purified with SakA and MpkC during osmotic and CR stresses are listed in Supplementary Tables S1, S2, respectively.

**FIGURE 1:**
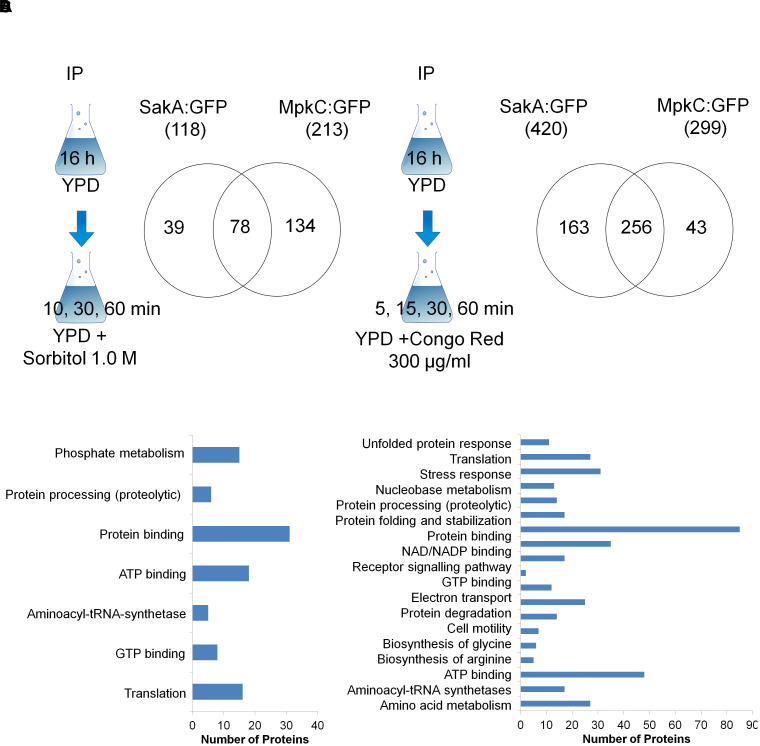
Scheme and Venn diagram of the pull-down experiments for proteins that interact with SakA:GFP and MpkC:GFP upon osmotic **(A)** and cell wall stresses **(B)**. A summary of the FunCat terms over-represented (adjusted *p*-value < 0.05) for proteins observed as interacting with SakA:GFP and MpkC:GFP upon osmotic **(C)** and cell wall stresses **(D)**. For the full list refer to Supplementary Tables S1–S10.

In presence of sorbitol, 118 and 213 proteins were detected as possible protein interactors of SakA and MpkC, respectively. Interestingly, a group of 78 proteins were common to both protein interactomes (Figure 1A). Under CR stress, 420 and 299 proteins were detected interacting with SakA and MpkC, respectively (Figure 1B). Once more a common group of 256 proteins were identified in both interactomes (Figure 1B) which strongly suggests that SakA and MpkC have some common functions in *A. fumigatus*. Among the proteins considered associated with MpkC (Afu5g09100), SakA (Afu1g12940) was identified under CR and osmotic stresses (Supplementary Tables S9, S10), suggesting that MpkC is a SakA interactor. To verify possible interaction networks, the proteins identified by proteomics were analyzed using STRING^1^. We were able to identify about 72.0–87.8% of the immunoprecipitated proteins as biologically interacting with either MpkC:GFP, SakA:GFP, both proteins or proteins that interact with them (Supplementary Tables S11, S12; osmotic and CR: MpkC:GFP, 80.3, and 85.3%; SakA:GFP, 72.0 and 84.3%; and MpkC:GFP and SakA:GFP, 79.2 and 87.8%).

FunCat^2^ enrichment analyses for both strains under osmotic stress demonstrated an enrichment for proteins involved in phosphate metabolism, protein processing, ATP and GTP binding, and translation (osmotic stress, Figure 1C). FunCat for both strains upon CR stress showed an enrichment for proteins involved in unfolded protein response, translation, stress response, protein processing, electron transport, biosynthesis of glycine and arginine, and amino acid metabolism (Figure 1D). This implies that SakA and MpkC collaborate in several biological processes involving stress responses, translation and protein modification, and amino acid metabolism upon osmotic and cell wall stresses.

### SakA and MpkC Interact to Regulate Cell Membrane and Wall Biogenesis During Stress

Among the proteins identified in the SakA or MpkC interactome there are potential partners that can be associated to the functions of these kinases. These comprise MpkA (Afu4g13720, Tables 2, 4), the central regulator of CWI pathway, whose phosphorylation during cell wall, and osmotic stresses is regulated by SakA (de Oliveira Bruder Nascimento et al., 2016), and PtcB (Afu1g09280, Table 3), a putative HOG phosphatase involved in regulation of MpkA and SakA phosphorylation (Bom et al., 2015). PakA (Afu2g04680, Table 3), found to be associated with SakA, is the putative homolog of *S. cerevisiae* Ste20, which is involved in the response to osmotic stress (Tanaka et al., 2014). Other proteins that co-purified with SakA or MpkC include some proteins involved in the biosynthesis of the cell wall polysaccharides (Afu3g12690, Afu7g02180, Afu6g12400, Afu1g06210, Afu2g05340, Afu7g05450, and Afu3g14420, Tables 1–4) and ergosterol (Afu3g10660, Afu6g14200, Afu5g02450, Afu7g03740, Afu4g06890, Afu4g07130, and Afu4g03630, Tables 1–3). The transcription factors DvrA (Afu3g09820, Table 1), the putative *C. albicans* Bcr1p ortholog, that regulates biofilm formation and expression of cell-surface genes, and NsdD (Afu3g13870, Tables 1, 3, 4), required during an early stage of mating, that plays a role in resistance toward cell wall stress, were also identified in the SakA interactome. In response to osmotic and cell wall stresses, SakA and MpkC were also found to be associated with some heat shock proteins and chaperones (Afu2g02320, Afu7g01860, Afu1g06710, Afu1g01740, Afu6g07540, Afu7g01860, Afu3g14540, Afu5g13920, and Afu2g16020, Tables 1–4). We observed the interaction of these MAPK with the glucan synthase Fks1 (Afu6g12400), which facilitates the production of the major cell wall component 1,3 β-D-glucan, Agm1 (Afu1g06210), a N-acetylphosphoglucosamine mutase involved in chitin biosynthesis, Gel4 (Afu2g05340), a essential 1,3 β-glucanosyltransferase and Sun1 (Afu7g05450), a 1,3 β-glucan modifying enzyme involved in fungal morphogenesis (Tables 1–4). SakA and MpkC also interact with proteins involved in the ergosterol biosynthetic pathway including the HMG-CoA synthase Erg13 (Afu3g10660) and the sterol demethylase proteins, Cyp51A (Afu4g06890) and Cyp51B (Afu7g03740), that are related to the mechanisms for azole drug resistance (Tables 1–4). These proteins are possibly related to SakA and MpkC functions.

**Table 1:** Selected proteins interacting with SakA::GFP identified during osmotic stress.

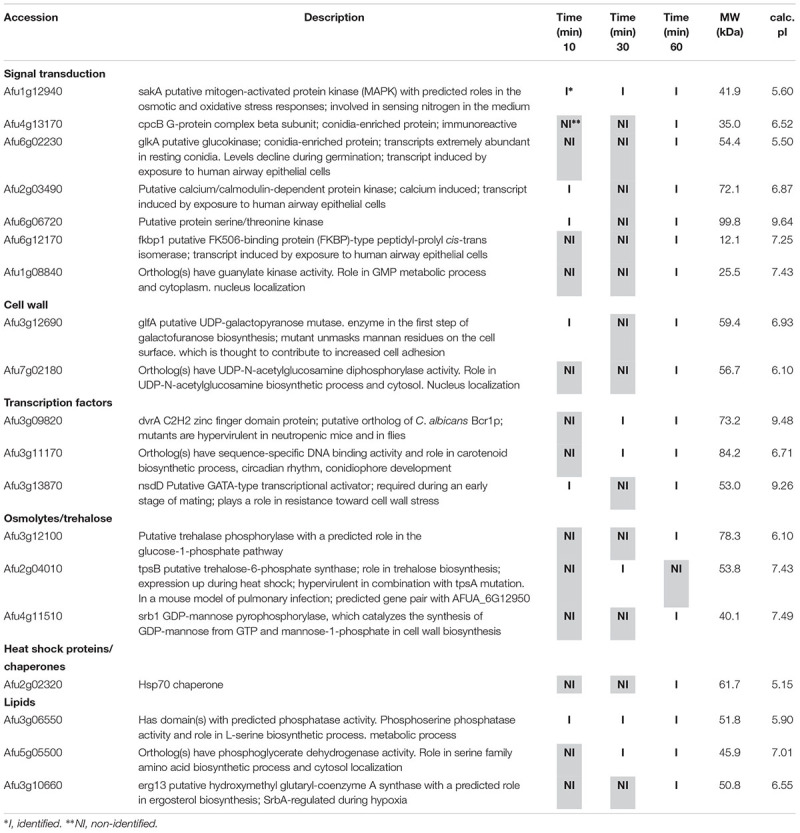

**Table 2:** Selected proteins interacting with MpkC::GFP identified during osmotic stress.

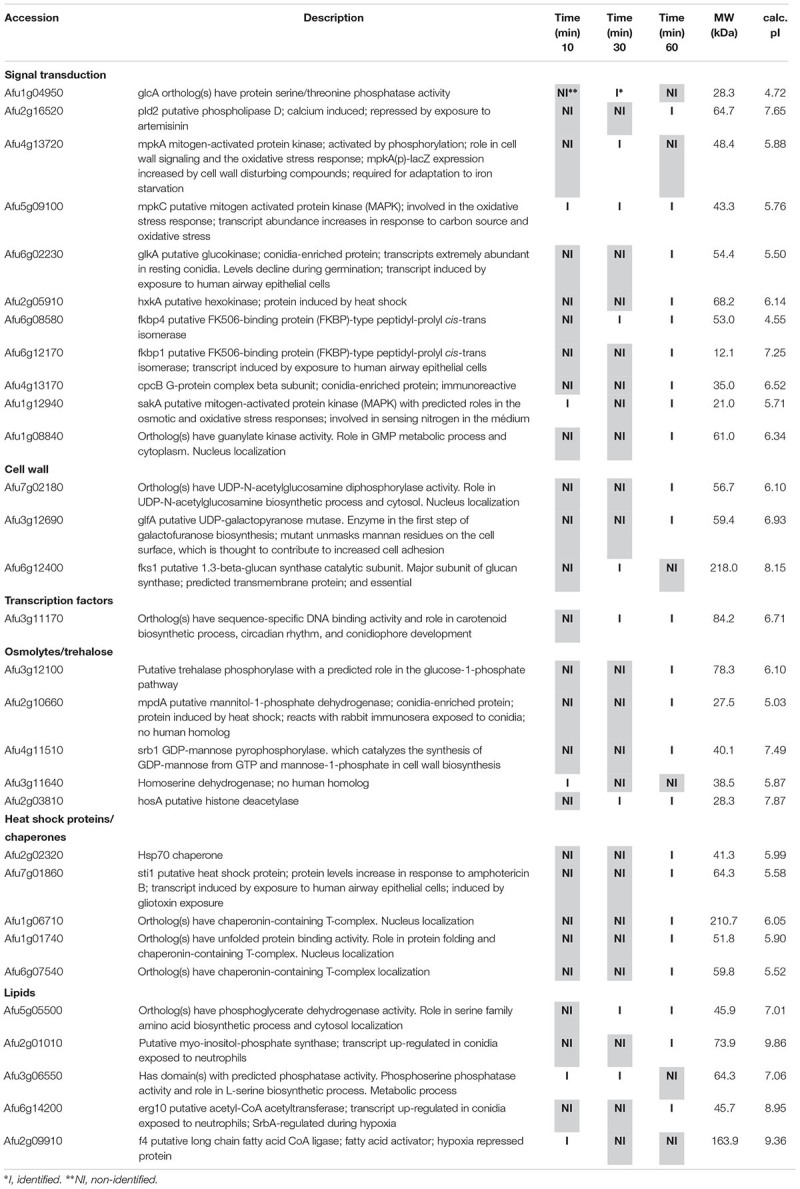

**Table 3:** Selected proteins interacting with SakA::GFP identified during cell wall stress.

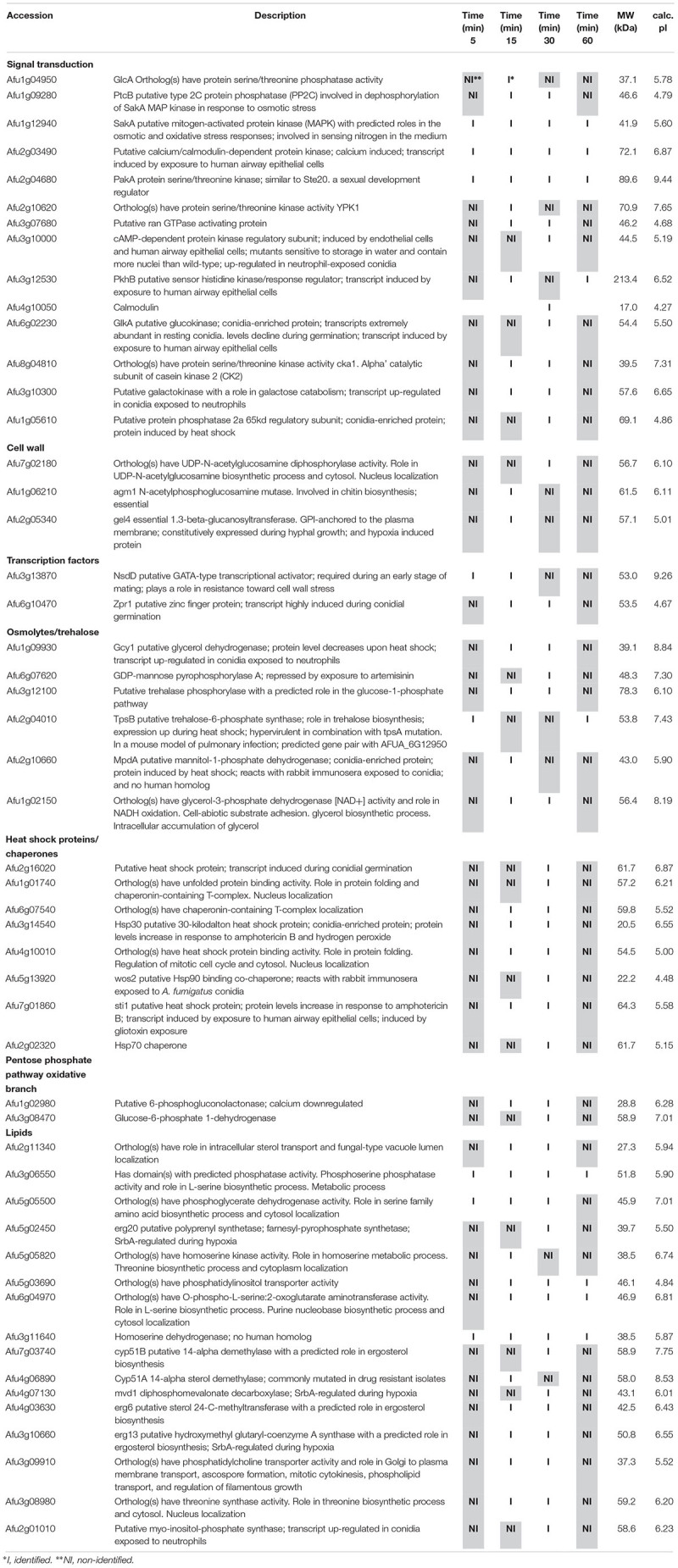

**Table 4:** Selected proteins interacting with MpkC::GFP identified during cell wall stress.

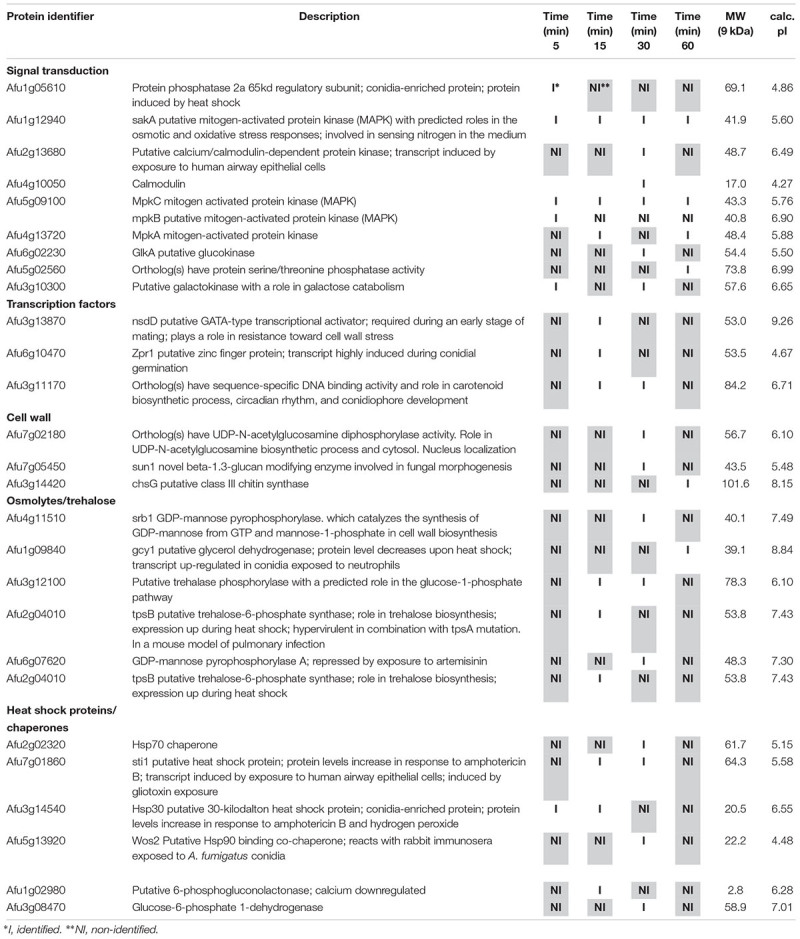

In summary, SakA and MpkC show both physical and functional interactions and that SakA/MpkC pathways play important roles in the signaling processes that regulate the response to osmotic stress and cell wall damage.

### SakA and MpkC Physically Associate During Osmotic and Cell Wall Stresses

**FIGURE 2:**
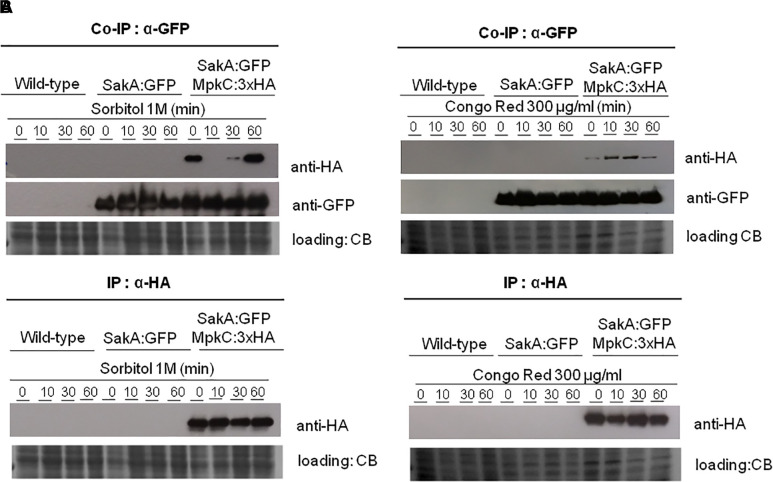
Co-immunoprecipitation (Co-IP) of SakA:GFP and MpkC:3xHA. **(A)** Verification of association between SakA:GFP and MpkC:3xHA by Co-IP. Affinity purification assays from GFP-tagged SakA strain in the background of 3xHA-tagged MpkC were performed with GFP-Trap and anti-HA beads to verify interactions upon sorbitol **(A)** and cell wall stresses **(B**). The coimmunoprecipitated proteins were analyzed by the indicated antibodies. CB, coomassie blue staining.

To confirm the physical association between SakA and MpkC we decide to carry out co-immunoprecipitation (Co-IP) experiments (see section “Materials and Methods”) using protein extracts from wild-type and strains expressing SakA:GFP and SakA:GFP MpkC:3xHA. We observed no changes in the phenotypes of these tagged strains compared to the wild-type (de Oliveira Bruder Nascimento et al., 2016; Supplementary Figure S1). Protein extracts were prepared from wild-type, SakA:GFP and SakA:GFP MpkC:3xHA strains that were untreated or treated with sorbitol 1.0 M or CR 300 μg/ml for 10, 30 and 60 min. Figure 2 shows that pull-down of SakA:GFP results in co-purification of MpkC only in the strain expressing SakA:GFP MpkC:3xHA and not in a wild-type or SakA:GFP strains. Moreover, this interaction occurs even in the absence of stress conditions (without Sorbitol and CR). We also tried to IP MpkC:3xHA and SakA:GFP first before probing for MpkC with an anti-HA or anti-GFP antibody, but the MpkC:3xHA and SakA:GFP tags interacted with the IP resin in control conditions, therefore de-validating the assay (data not shown).

### SakA Associates With the MAPK MpkA and the Protein Phosphatase PtcB

**FIGURE 3:**
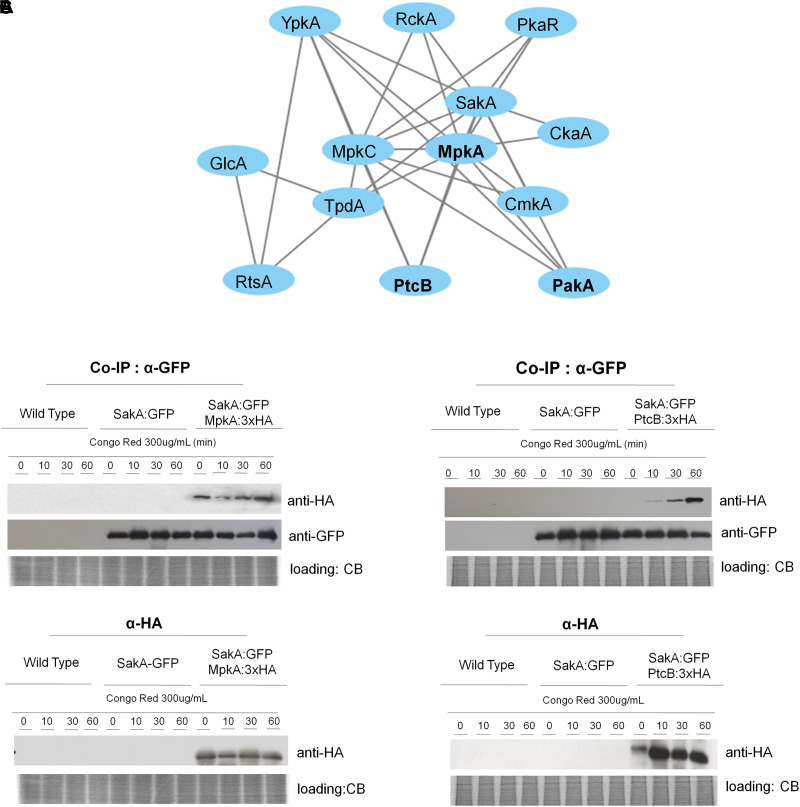
SakA:GFP associates with MpkA and PtcB. **(A)** Interaction network performed by using STRING (https://string-db.org/) showing the interaction among SakA, MpkC, MpkA, PtcB, and PakA. **(B)** Co-IP of SakA:GFP, MpkA:3xHA, SakA:GFP, and PtcB:3xHA. Affinity purification assays from GFP-tagged SakA strain in the background of 3xHA-tagged MpkA or PtcB were performed with GFP-Trap and anti-HA beads to verify interactions upon cell wall stress. CB, coomassie blue staining.

As both MpkA and PtcB have been implicated in the HOG and CWI pathway and here they were identified in the SakA interactome in presence of CR (Figure 3A and Tables 2, 4), we used pull-down experiments to validate these interactions. We introduced into SakA:GFP strain, a plasmid expressing MpkA or PtcB tagged at C-terminus with HA tag. We showed that HA-tagging did not affect the phenotypes of these strains compared to the wild-type (Supplementary Figures S2, S3). We carried out Co-IP assays using GFP-Trap beads with the wild-type, SakA:GFP, SakA:GFP MpkA:3xHA (Figure 3B) and SakA:GFP PtcB:3xHA (Figure 3C) that were untreated or treated with CR 300 μg/ml (10, 30, and 60 min). Interaction of HA tagged MpkA with PtcB were then identified with HA antibody via Western blot. The results of the immunoblotting analysis showed that MpkA and PtcB were only co-immunoprecipitated from the SakA:GFP MpkA:3xHA (Figure 3B) and SakA:GFP PtcB:3xHA (Figure 3C), respectively, illustrating that these two protein associate with SakA. Moreover, our results show that SakA-PtcB interaction occurs only in presence of CR stress (Figure 3C). We also tried to IP MpkA:3xHA or PtcB:3xHA and SakA:GFP first before probing for MpkA or PtcB with an anti-HA or anti-GFP antibody, but once more the MpkA:3xHA or PtcB:3xHA and SakA:GFP tags interacted with the IP resin in control conditions, therefore de-validating the assay (data no shown). All together, these data show that the interactions found in the SakA and MpkC interactomes could be robustly validated using Co-IP approach.

### Molecular Characterization of PakA^STE20^

**FIGURE 4:**
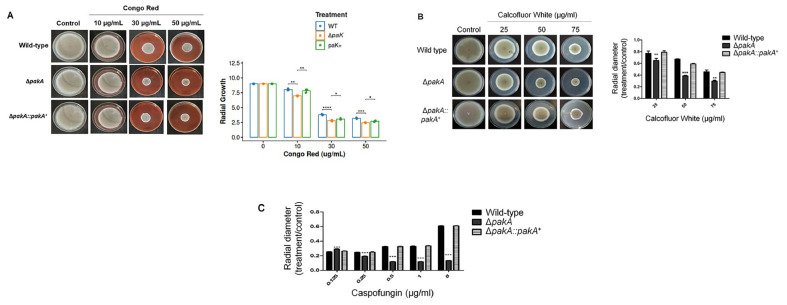
PakA is important for the response to cell wall stress. **(A)** The wild-type, ΔpakA, and ΔpaKA:pakA+ and ΔcrzA ΔzipD mutant strains were grown on minimal media with increasing concentrations of congo red **(A)**, calcofluor white (CFW) **(B)**, and caspofungin **(C)** for 5 days at 37 oC. The results are expressed as the average of three repetitions ± standard deviation. Statistical analysis was performed using a one-way ANOVA test when compared to the wild-type condition (^∗∗^*p* < 0.005; ^∗∗∗^*p* < 0.001).

Here we identified PakA, the homolog of the *S. cerevisiae* sexual developmental serine/threonine kinase (Afu2g04680) Ste20, as a component of the SakA/MpkC MAPK pathway. To gain an insight into the function of the Ste20 homolog in *A. fumigatus*, a *pakA* null mutant, and complemented strains were constructed (Supplementary Figure S4). The wild-type, *ΔpakA* and *ΔpakA*:*pakA*^+^ strains were grown in minimal medium (MM) and exposed to agents that affect CWI pathway, including CR, calcofluor white (CFW), and the echinocandin caspofungin (Figure 4A–C). The *ΔpakA* strain showed radial growth similar to the wild-type strain in MM (Figure 4). The *ΔpakA* strain was slightly more sensitive to CR (Figure 4A) and showed similar sensitivity to high sorbitol concentrations when compared to the wild-type strain (data not shown). The *ΔpakA* was sensitive to CFW and caspofungin stresses (Figure 4B,C), and the loss of the caspofungin paradoxical effect (CPE, a phenomenon where high caspofungin concentrations revert the anticipated inhibition of *A. fumigatus* growth; Steinbach et al., 2015; Figure 4C).

**FIGURE 5:**
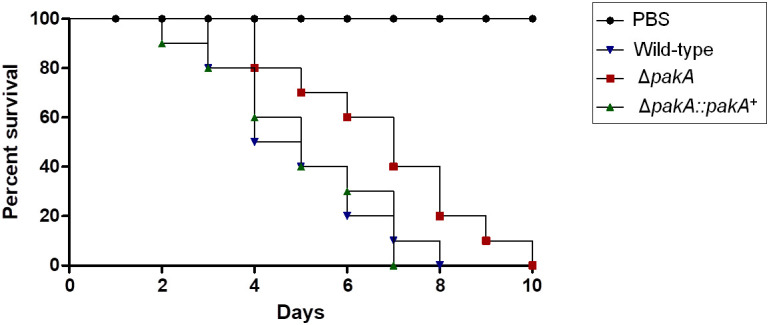
*Aspergillus* *fumigatus* PakA contributes to virulence in *G. mellonella*. Comparative analysis of wild-type and mutant strains in *G. mellonella* animal model. Larvae in groups of 10 per strain were infected with a 5 μl suspension of conidia at a dose of 1 × 106/larva. PBS, phosphate buffer saline.

Finally, to further characterize the possible effect of PakA on pathogenicity of *A. fumigatus*, an *Galleria mellonella* model which had been demonstrated as a good model to evaluate fungal pathogenicity was used (Slater et al., 2011). In the *G. mellonella* model, infection by either of wild-type or the complementing strains resulted in 100% mortality 8 and 7 days post-infection, respectively (Figure 5). However, the Δ*pakA* mutant strain showed 100 % mortality 10 days post-infection, which was statistically different to the wild-type, and complementing strains according to the Mantel-Cox and Gehan-Brestow-Wilcoxon tests (*p*-values 0.0041 and 0.0124, respectively). These results suggest that the lack of *pakA* attenuated the *A. fumigatus* virulence in this animal model.

## Discussion

Fungi live in diverse environments ranging from soil to mammalian host. Fungi are also exposed to many stressing conditions including heat shock, oxidative stress, osmotic and pH changes, chemical challenges, and nutrient limitations. These stress conditions can be found either in natural habitats or in animal and human hosts during the infection process (Brown and Goldman, 2016). Exposure of fungal cells to these stress conditions leads to the activation of some MAPK cascades (Pearson et al., 2001; Rispail et al., 2009). In fungi the signaling through the MAPK cascades result in altered gene expression that regulates many processes including pheromone response, filamentous growth, biosynthesis of cell wall components, the HOG pathway, the establishment of virulence, and mediation of drug resistance (Bahn et al., 2005; Monge et al., 2006; Román et al., 2007; Valiante et al., 2008, 2009, 2015a,b; de Castro et al., 2014; Altwasser et al., 2015; Bom et al., 2015; Winkelströter et al., 2015a,b).

In *A. fumigatus*, Hog1 orthologs SakA and MpkC show multifunctional roles. In addition to the role played in the osmotic stress response, these MAPKs also regulate stress response to stimuli such as oxidative stress, cell wall damaging agents (de Oliveira Bruder Nascimento et al., 2016) and also play a role in caspofungin adaptation, carbon source utilization, and collaborate during virulence establishment (Reyes et al., 2006; Altwasser et al., 2015; Valiante et al., 2015a; de Oliveira Bruder Nascimento et al., 2016). *A. nidulans* SakA and MpkC not only physically interact, but also show opposite and common functions during stress responses and development (Jaimes-Arroyo et al., 2015; Garrido-Bazán et al., 2018). However, little is known about the mechanisms by which SakA and MpkC execute their signaling functions.

We have identified by mass spectrometry many common SakA and MpkC protein interactors, suggesting they perform similar roles. Pull-down experiments strongly indicated SakA and MpkC are physically interacting, possibly forming a protein complex which regulates these down stream processes under specific conditions. Subsequently, by using SakA GFP-tag pull-down and mass spectrometry analysis, we showed that SakA interacts not only with MpkC, but also with the CWI pathway MAPK MpkA, the HOG response phosphatase PtcB and other proteins involved in signal transduction pathways, biosynthesis of the cell wall, transcription factors, heat shock proteins, and chaperones. Previously, we proposed that SakA and MpkC are interacting and that MpkC could be a modulator of SakA during HOG and CWI pathways, since SakA appears to play a major role in response to several types of stresses (de Oliveira Bruder Nascimento et al., 2016). In this work we proved this interaction by using Co-IP experiments and MpkC was showed to be continuously associated with SakA (stress and no stress). This resembles what happens in *A. nidulans* where MpkC was found to be linked with SakA with and without oxidative stress (Jaimes-Arroyo et al., 2015). Furthermore, we showed previously that both MpkC:GFP and SakA:GFP translocated to the nucleus upon osmotic stress, with SakA:GFP showing a quicker response (de Oliveira Bruder Nascimento et al., 2016). This data can explain the absence of MpkC co-purified with SakA after 10 min of sorbitol exposure. Our pull-down experiments not only showed SakA-MpkC interaction, but also identified a common group of 78 and 256 proteins that potentially interact with both SakA and MpkC upon osmotic stress and cell wall damage, respectively. It is clear that these common interactions confirm that SakA-MpkC interact with similar proteins in *A. fumigatus*.

Previous work showed that the phosphorylation level of the MpkA protein during osmotic stress and cell wall damage is regulated by SakA and MpkC. The *ΔsakA* mutant showed reduced MpkA phosphorylation and the double *ΔmpkC ΔsakA* demonstrated no detectable MpkA phosphorylation (de Oliveira Bruder Nascimento et al., 2016). Furthermore, Altwasser et al. (2015) using systems biology approach, demonstrated the activation and cross talk between the *A. fumigatus* MpkA and SakA pathways during treatment with increased caspofungin doses. These authors have demonstrated that after exposure to caspofungin there is a higher level of SakA phosphorylation in the *ΔmpkA* mutant. Additionally, caspofungin influenced intracellular transport inducing a further osmotic stress; however this osmotic stress is reduced under high concentrations of caspofungin (Chen et al., 2011). The data presented here shows that MpkA and SakA are interactive under unstressed conditions and during the cell wall stress. In addition, our data show that SakA and MpkC associate with some proteins involved in the biosynthesis of the cell wall during the presence of osmotic stress and cell wall damage. Collectively, these presented data indicate that SakA and MpkC are allowing the signal integration and information exchange between HOG and CWI pathways.

Among the proteins identified in the Co-IP experiments that might be directly related to SakA function include the phosphatase PtcB. Previously we identified PtcB as a phosphatase related to the HOG pathway. We have shown that the *ΔptcB* strain has both increased phosphorylation of SakA and MpkA, and regulates the expression of osmo-dependent genes (Winkelströter et al., 2015a). Our results provide the new insights into the mechanisms by which PtcB influences the HOG pathway in *A. fumigatus*.

Our results show that *A. fumigatus* mutant lacking the kinase PakA, homolog of the *S. cerevisiae* Ste20, is sensitive to agents that affect CWI pathway, and PakA does not mediate the sensitivity to osmotic stress. In yeast, Ste20p is involved in cellular responses to nutritional limitation and mating pheromone, and is required to establish cell polarity (Cvrcková et al., 1995; Pringle et al., 1995; Simon et al., 1995; Richman et al., 1999). In addition, Ste20p is necessary for osmotic stress response via the Sho1p branch of the HOG pathway in *S. cerevisiae* (Raitt et al., 2000). Vaga et al. (2014) showed by phosphoproteomic analyses that yeast Ste20p is a key mediator of the Hog1 phosphorylation in response to high osmolarity and mating pathways. In this work, we reported the interaction between SakA and PakA. We then showed for the first time that PakA kinase is a component of the SakA/MpkC MAPK pathway in *A. fumigatus* which contributed to cell wall and caspofungin stress, plus virulence.

Together, our data support the hypothesis that SakA and MpkC are part of an osmotic and general signal pathways involved in regulation of the response to the cell wall damage, oxidative stress, drug resistance, and establishment of infection. The identification and characterization of novel protein interactors that are directly linked to SakA and MpkC function vital to antifungal resistance and virulence, represent potential new targets for the development of new antifungals combating IA.

## Materials and Methods

### Strains, Media, and Growth Conditions

All strains used in this study are listed in Supplementary Table S13 in the supplemental material, and the primers used are listed in Supplementary Table S14. The *A. fumigatus* parental strain were CEA17 (pyrG+) *akuBKU80* and CEA17 (pyrG-) *akuBKU80* (da Silva Ferreira et al., 2006). The MpkC:GFP and SakA:GFP strains were constructed by de Oliveira Bruder Nascimento et al. (2016). Media were of two basic types. A complete medium [YAG: 2% (w/v) glucose, 0.5% (w/v) yeast extract, 2% (w/v) agar, trace elements] with three variants: YUU (YAG supplemented with 1.2 g/l each of uracil and uridine) and liquid YG or YUU medium of the same composition but without agar. A modified minimal medium (MM: 1% (w/v) glucose, original high nitrate salts, trace elements, 2% (w/v) agar, pH 6.5) was also used. Trace elements, vitamins, and nitrate salts were described by Kafer (1977).

### Plasmid Constructions

All *A. fumigatus* genes were isolated from the strain CEA17 D’Enfert et al. (1996). The cassette for *pakA* deletion was constructed by *in vivo* recombination in *S. cerevisiae* as previously described by Colot et al. (2006). Thus, approximately 2.0 kb from the 5′-UTR and 3′-UTR flanking region of the targeted ORF regions was selected for primer design. The primers 5F (pakA pRS426 5fw) and 3R (pakA pRS426 3rv) contained a short homologous sequence to the MCS of the plasmid pRS426. Both the 5- and 3- UTR fragments were PCR amplified from *A. fumigatus* genomic DNA (gDNA). The pyrG gene placed within the cassette as a prototrophic marker was amplified from pCDA21 plasmid. The deletion cassette was generated by transforming each fragment along with the plasmid pRS426 cut with BamHI/EcoRI into the *S. cerevisiae strain* SC94721 using the lithium acetate method (Schiestl and Gietz, 1989). The DNA from the transformants was extracted by the method described by Goldman et al. (2003). The cassette was PCR amplified from these plasmids utilizing TaKaRa Ex Taq^TM^ DNA Polymerase (Clontech Takara Bio) and used for *A. fumigatus* transformation. Southern blot analysis demonstrated that the transformation cassette had integrated homologously at the targeted loci (Supplementary Figure S4). The single gene deletion of the *pakA* was complemented by co-transforming a DNA fragment (approximately 1 kb from each 5′ and 3′ flanking regions plus the ORF) together with the pHATα (Herrera-Estrella et al., 1990) and selecting for hygromycin resistance in MM plates with 250 mg/ml of hygromycin B. Southern blot was used to confirm the reconstituted strain (Supplementary Figure S4). For the generation of 3xHA fusion fragments mpkC:3xHA:ptrA, mpkA:3xHA:ptrA and ptcB:3xHA:ptrA, a portion of DNA consisting of the gene ORF and 5′ UTR region, along with a 1-Kb segment of DNA consisting of the 3′ UTR flanking region were amplified with the primers pairs listed in supplemental table SX, from CEA17 gDNA. The 0.8 kb *3xHA-trpC* fusion was amplified with primers OZG916/trpC REV ptrA from the pOB430 plasmid and a *ptrA* fragment amplified from the plasmid pPTR. The cassette was generated by transforming each fragment along with the plasmid pRS426 cut with BamHI/EcoRI into the *S. cerevisiae* strain. These cassettes were then transformed into the CEA17 strain and verification of 3xHA tagged strains were confirmed via PCR reaction (Supplementary Figures S1–S4).

Southern blot and PCR analyses were used to demonstrate that the cassettes had integrated homologously at the targeted *A*. *fumigatus* loci. Genomic DNA from *A*. *fumigatus* was extracted by grinding frozen mycelia in liquid nitrogen and then gDNA was extracted as previously described (Malavazi and Goldman, 2012). Standard techniques for manipulation of DNA were carried out as described (Sambrook and Russell, 2001). For Southern blot analysis, restricted chromosomal DNA fragments were separated on 1% agarose gel and blotted onto Hybond N+ nylon membranes (GE Healthcare). Probes were labeled using [α-32P]dCTP using the Random Primers DNA Labeling System (Life Technologies). Labeled membranes were exposed to X-ray films, which were scanned for image processing. Southern blot and PCR schemes are shown in Supplementary Figures S1–S4.

### Phenotypic Assays

The phenotypes of the deletion mutant *ΔpakA* were evaluated by radial growth in MM and in presence of agents that affect CWI, including CR, calcofluor white (CFW) and the echinocandin caspofungin. The experiments were performed using 5 μl of a 2 × 107 conidia for the wild-type and mutant strain and grown for 96 h at 37°C.

### Protein Interaction Network Analysis

For the analysis of the interaction networks, proteins identified by proteomics were analyzed using STRING^3^ considering medium confidence parameter, and all possible interaction parameters allowed (such as co-expression, experimental evidence, and co-occurrence, etc). Resulting interaction networks were further processed using *ad hoc* Perl scripts and plots were generated using Gephi^4^.

### GFP-Tag Protein Purification and Identification by LC-MS/MS

To precipitate GFP-tag-labeled SakA and MpkC, protein crude extracts were prepared from wild-type, SakA:GFP and MpkC:GFP cultures grown for 24 h and further exposed to sorbitol 1.0 M (10, 30, and 60 min) or CR 300 μg/mL (5, 15, 30, and 60 min). Crude protein extracts from mycelia were obtained by extraction from ground mycelia with B250 buffer (250 mM NaCl, 100 mM Tris–HCl pH 7.5, 10% glycerol, 1 mM EDTA and 0.1% NP-40) supplemented with 1.5 ml/L 1 M DTT, 2 tables/100 mL complete-mini protease inhibitor cocktail EDTA-free (Roche), 3 ml/L 0.5 M Benzamidine, 10 ml/L phosphatase inhibitors 100× (10 M NaF, 5 M Na Vanadate, 8 M β-glycerol phosphate), and 10 ml/L 100 mM PMSF. Total protein lysates were submitted to centrifuge at 13.000 rpm at 4°C for 10 min, the supernatant was collected into a new eppendorf. Magnetics GFP-trap beads were equilibrated with lysis buffer B250 (20 uL of beads into 500 uL lysis buffer B250; de Assis et al., 2018) during 10 min, after then were collected using magnet hack and incubated with total protein lysate at 4°C during 3 h. After incubation the magnetics GFP-trap beads were collected using magnetic hack and the supernatant was removed. The magnetic GFP-trap beads were washed two times using 500 uL lysis buffer B250 without DTT and one additional wash step was done with addition of DTT. The magnetics GFP-trap beads were collected and supernatant was removed. The LC-MS/MS identification was performed as described previously (Johnk et al., 2016). Digested peptides were separated using reversed-phase liquid chromatography with an RSLCnano Ultimate 3000 system (Thermo Fisher Scientific) followed by mass identification with an Orbitrap Velos Pro mass spectrometer (Thermo Fisher Scientific). Chromatographically separated peptides were on-line ionized by nano-electrospray (nESI) using the Nanospray Flex Ion Source (Thermo Fisher Scientific) at 2.4 kV and continuously transferred into the mass spectrometer. Full scans within m/z of 300–1850 were recorded by the Orbitrap-FT analyzer at a resolution of 30.000 (using m/z 445.120025 as lock mass) with parallel data-dependent top 10 MS2-fragmentation in the LTQ Velos Pro linear ion trap. LCMS method programming and data acquisition was performed with the software XCalibur 2.2 (Thermo Fisher Scientific) and method/raw data validation with the program RawMeat 2.1 (Vast Scientific). MS/MS2 data processing for protein analysis and identification was done with either MaxQuant quantitative proteomic software in conjunction with Perseus software for statistical analysis or the Proteome Discoverer 1.3 (PD, Thermo Fisher Scientific) and the Discoverer Daemon 1.3 (Thermo Fisher Scientific) software using the Sequest (and/or Mascot) peptide analysis algorithm(s) and organism-specific taxon-defined protein databases extended by the most common contaminants.

### Co-IPs With GFP-Trap and Anti-HA Magnetic Beads

To perform co-IP assays, C-terminal HA-tagged MpkC, MpkA, and PtcB strains were generated in the SakA:GFP background. The strains were grown for 16 h in MM and further exposed to sorbitol 1.0 M (10, 30, and 60 min) or CR 300 μg/mL (5, 15, 30, and 60 min). GFP-Trap co-IP experiments were performed as previously reported (Manfiolli et al., 2017). To perform reciprocal coimmunoprecipitation assays mycelia were frozen with liquid nitrogen, ground, and 500 mg was resuspended in 1 ml of B250 buffer (see above). Samples were centrifuged at 16,100 ×*g* for 10 min at 4°C. Supernatant was collected, and a Bradford assay (BioRad) was carried out to measure protein content. The same amount of protein for each sample was added to 20 uL of Dynabeads Protein A (Thermo Fisher Scientific) previously incubated with monoclonal anti-HA antibody (Sigma). The resin was washed three times with resuspension buffer prior to incubation. Cell extracts and resin were then incubated with shaking at 4°C for 2 h. After incubation, the resin was washed three times in resuspension buffer by placing the tube in a DynaMag^TM^ magnet. To release the proteins from the resin, samples were incubated with Sample Buffer and boiled at 98°C for 5 min. Proteins were transferred from a 10% SDS-PAGE gel onto a nitrocellulose membrane for a Western blot assay using a *Trans*-Blot turbo transfer system (Bio-Rad). GFP-tagged SakA was detected using a rabbit anti-GFP antibody (Abcam) at 1:2,000 dilution and a goat anti-rabbit I gG horseradish peroxidase (HRP) antibody (Cell Signaling Technology) at 1:10,000 dilution. For the HA-tagged proteins detection, a mouse monoclonal anti-HA antibody (Sigma) was used at 1:2,000 dilution as a primary antibody followed by an anti-mouse I gG HRP conjugate (Cell Signaling Technology) used at 1:10,000 dilution as a secondary antibody.

### *Galleria mellonella* Experiments

*Galleria mellonella* larvae were obtained by breeding adult moths (Fuchs et al., 2010). *G. mellonella* larvae of a similar size were selected (approximately 275–330 mg) and kept without food in glass container (Petri dishes), at 37°C, in darkness for 24 h prior to use. *A. fumigatus* conidia were obtained by growing on YAG media culture for 2 days. The conidia were harvested in PBS and filtered through a Miracloth (Calbiochem). The concentration of conidia was estimated by using hemocytometer, and resuspended at a concentration of 2.0 × 10^8^ conidia/ml. The viability of the conidia was determined by incubating on YAG media culture, at 37°C, 48 h. Inoculum (5 μl of a 2 × 10^8^ conidia/ml) from the wild-type, mutant and complemented strains (1 × 10^6^ conidia/larva) were used to investigate the virulence of *A. fumigatus* against *G. mellonella*. Ten *G. mellonella* in the final (sixth) instar larval stage of development were used per condition in all assays. The control group was the larvae inoculated with 5 μl of PBS to observe the killing due to physical trauma. The inoculum was performed by using Hamilton syringe (7000.5KH) and 5 μl into the haemocel of each larva via the last left proleg. After, the larvae were incubated in glass container (Petri dishes) at 37°C in the dark. The larval killing was scored daily. Larvae were considered dead by presenting the absence of movement in response to touch.

## Author Contributions

AM and NB wrote the manuscript. AM, EM, LA, LS, RS-R, and MU performed the experiments. NB and GG reviewed the manuscript. ÖB and GG designed the experiments and obtained the financial resources.

## Conflict of Interest Statement

The authors declare that the research was conducted in the absence of any commercial or financial relationships that could be construed as a potential conflict of interest.

## Footnotes

1
https://string-db.org/

2
https://bio.tools/fungifun

3
https://string-db.org/

4
https://gephi.org/
