# Correction: *Aspergillus fumigatus* high osmolarity glycerol mitogen activated protein kinases SakA and MpkC physically interact during osmotic and cell wall stresses

**DOI:** 10.3389/fmicb.2026.1893398

**Published:** 2026-06-23

**Authors:** Adriana Oliveira Manfiolli, Eliciane Cevolani Mattos, Leandro José de Assis, Lilian Pereira Silva, Mevlüt Ulaş, Neil Andrew Brown, Rafael Silva-Rocha, Özgür Bayram, Gustavo H. Goldman

**Affiliations:** 1 Faculdade de Ciências Farmacêuticas de Ribeirão Preto, Universidade de São Paulo, Ribeirão Preto, Brazil; 2 Department of Biology, Maynooth University, Maynooth, Ireland; 3 Department of Biology and Biochemistry, University of Bath, Bath, United Kingdom; 4 Faculdade de Medicina de Ribeirão Preto, Universidade de São Paulo, Ribeirão Preto, Brazil

**Keywords:** *Aspergillus fumigatus*, mitogen activate protein kinase, HOG, MPKC, SakA

There was a mistake in Figure 4A as published.

By accident, the same image of the wild-type and complementing mutant strain was used at 50 μg/ml Congo Red. The experiment was repeated, and the corrected Figure 4A appears below.

**Figure 4:**
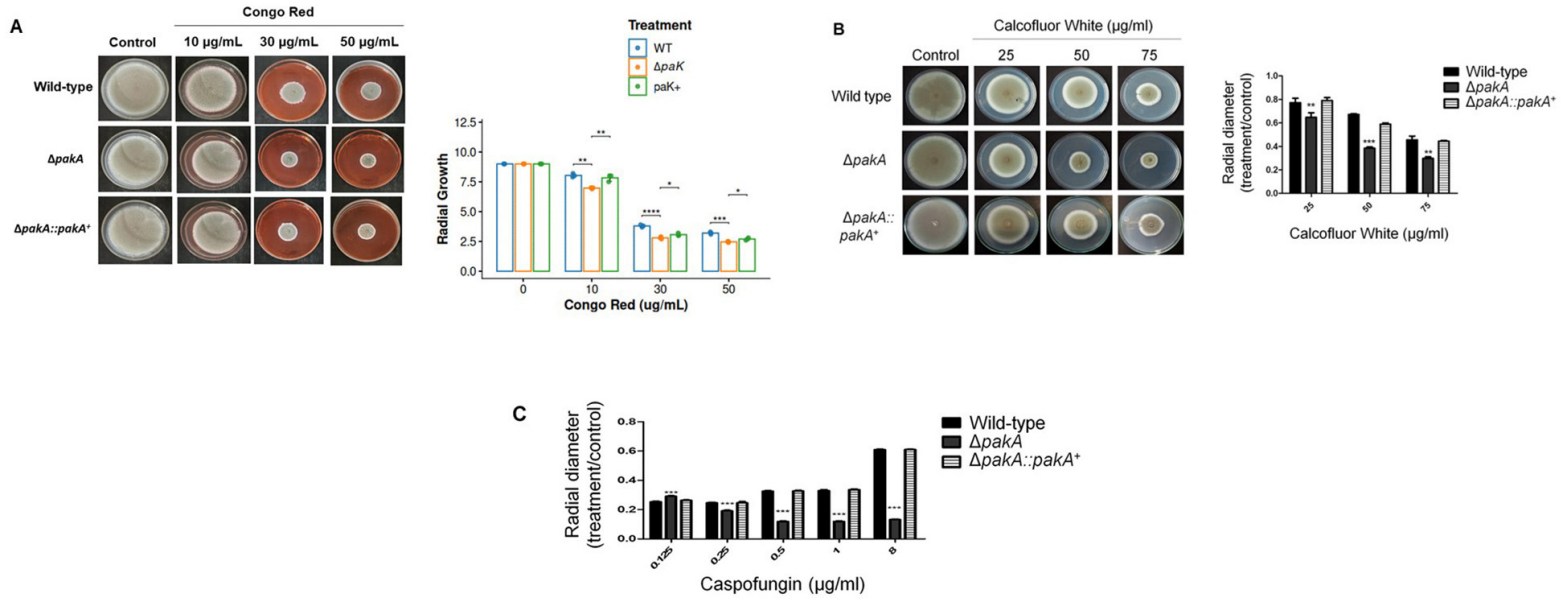
PakA is important for the response to cell wall stress. **(A)** The wild-type, ΔpakA, and ΔpaKA:pakA+ and ΔcrzA ΔzipD mutant strains were grown on minimal media with increasing concentrations of congo red **(A)**, calcofluor white (CFW) **(B)**, and caspofungin **(C)** for 5 days at 37 °C. The results are expressed as the average of three repetitions ± standard deviation. Statistical analysis was performed using a one-way ANOVA test when compared to the wild-type condition (^**^p < 0.005; ^***^p < 0.001).

The original version of this article has been updated.

