# Supplementary material for: Aspergillus fumigatus High Osmolarity Glycerol Mitogen Activated Protein Kinases SakA and MpkC Physically Interact During Osmotic and Cell Wall Stresses
Source: Front Microbiol. 2019 May 7;10:918. doi: 10.3389/fmicb.2019.00918 (PMC6514138; doi:10.3389/fmicb.2019.00918)
Supplement: Supplementary file 13 [file Table_13.docx]

**Supplementary Table S13. Strains used in this study.**

| Name | Genotype | Source |
| --- | --- | --- |
| \| CEA17 \|  \| \| --- \| --- \| | Wild-type strain | D’Enfert *et al.* (1996) |
| \| Ku80 \|  \|  \| \| --- \| --- \| --- \| | Δ*akuB*ku80*:: pyrG*AF | FGSCA1151 |
| \| Ku80pyrG \|  \|  \| \| --- \| --- \| --- \| | Δ*akuB*ku80*::pyrG*AF *pyrG-* | da Silva Ferreira *et al.* (2006) |
| SakA::GFP | ∆*akuB*;*sakA*::*sakA-gfp-hph*; Hyg^R^ | de Oliveira Bruder Nascimento *et al.* (2016) |
| MpkC::GFP | ∆*akuB*;*mpkC*::*mpkC-gfp-hph*; Hyg^R^ | de Oliveira Bruder Nascimento *et al.* (2016) |
| SakA::GFP MpkC::3xHA | ∆*akuB*;*sakA*::*sakA-gfp-hph*; Hyg^R^*; mpkC*::*ptrA* | This study |
| SakA::GFP MpkA::3xHA | ∆*akuB*;*sakA*::*sakA-gfp-hph*; Hyg^R^*; mpkA*::*ptrA* | This study |
| SakA::GFP PtcB::3xHA | ∆*akuB*;*sakA*::*sakA-gfp-hph*; Hyg^R^*; ptcB*::*ptrA* | This study |
