# Supplementary material for: Aspergillus fumigatus High Osmolarity Glycerol Mitogen Activated Protein Kinases SakA and MpkC Physically Interact During Osmotic and Cell Wall Stresses
Source: Front Microbiol. 2019 May 7;10:918. doi: 10.3389/fmicb.2019.00918 (PMC6514138; doi:10.3389/fmicb.2019.00918)
Supplement: Supplementary file 14 [file Table_14.docx]

**Supplementary Table S14. Primers used in this study.**

| Name | Sequence 5’-3’ |
| --- | --- |
| pakA pRS426 5fw | GTAACGCCAGGGTTTTCCCAGTCACGACGCAAATCAGCTACCGCCAAAAAAAAG |
| pakA pRS426 3rv | CGGTTAACAATTTCTCTCTGGAAACAGCAGCAGGGCAGGGGGACAGGCC |
| pyrG Fw | GCATGCAAGCTTGGCGTATTCTGTCTGAGAGGAGGC |
| pyrG Rv | GAATTCGCCTCAAACAATGCTCTTCACC |
| Hygro fw | ACCCCATAACAATTTCACACACGA |
| Hygro pRS426 3rv | GCGGTTAACAATTTCTCTCTGGAAACAGCCGCCAGGGTTTTCCCAGTCACGAC |
| OZG916 | GGAGGTGGTAGCGGTGGT |
| trpC REV prtA | CAATTGCCCGTCTGTCAGATCTGTAAAAAAGTTTCGGCC |
| mpkC pRS426 5fw (3xHA) | GTAACGCCAGGGTTTTCCCAGTCACGACGTTGGACGGCTGATTACCT |
| mpkC orf LINKER 3HA rv | AGGAACATCATAGGGATAACCACCGCTACCACCTCCGATTTGGCCCGAATCACT |
| mpkC 3utr prtA 3fw | CGGCTCATCGTCACCCCATGATAGTCCAACGCTGCTCGGTTG |
| mpkC pRS426 3rv | GCGGATAACAATTTCACACAGGAAACAGCTCATTTTCTAAACTGCAATCAAA |
| mpkC 5' ext | TTGTCGCCCTCCACTTAC |
| mpkA pRS426 5fw (3xHA) | ttgtaaaacgacggccagtgCGGCTACACACGACACTC |
| mpkA orf LINKER 3HA rv | taccacctccTTGGACATCCATCCCCCCATGATAGTGACAATAGTTCATTTGCGTGTTC CCG |
| mpkA 3utr prtA 3fw | tctcagacagACGTTGACTTTCGTATGAAG |
| mpkA pRS426 3rv | atccccgggtaccgagctcgGAAATCCGCTTCAACACC |
| mpkA 5' ext | AGGATCATCGATGGAGTG |
| ptcB pRS426 5fw (3xHA) | GTAACGCCAGGGTTTTCCCAGTCACGACGTTTGCTACAGTTACGGCTG |
| ptcB orf LINKER 3HA rv | taccacctccCGAGGAGGCAGACTTCTC |
| ptcB 3utr prtA 3fw | CCCATGATAGTGACAATAGTTCATTTGCGTGTTC |
| ptcB pRS426 3rv | GCGGATAACAATTTCACACAGGAAACAGCGCCATGAGGGGGTGTAATTTTAC |
| ptcB 5' ext | GTACCGCCACTTAAGAGG |
| pakA pRS426 5fw (3xHA) | GTAACGCCAGGGTTTTCCCAGTCACGACGGCTTTCCCCCACCTTTTTCC |
| pakA orf LINKER 3HA rv | TACCACCTCCAGCGCCCCCTTTCTGAGC |
| pakA 3utr prtA 3fw | CCCATGATAGTCTCTTCTCAACTGGCTGTCTTTCTATTTGTG |
| pakA pRS426 3rv | GCGGATAACAATTTCACACAGGAAACAGAGCAGGGCAGGGGGACAG |
| pakA 5' ext | TCCTCCTTGTCATCCTCT |
| trpC prtA fw | GGCCGAAACTTTTTTACAGATCTGACAGACGGGCAATTG |
| prtA rv | CTATCATGGGGTGACGATGAGCCG |
