# Supplementary material for: Aspergillus fumigatus High Osmolarity Glycerol Mitogen Activated Protein Kinases SakA and MpkC Physically Interact During Osmotic and Cell Wall Stresses
Source: Front Microbiol. 2019 May 7;10:918. doi: 10.3389/fmicb.2019.00918 (PMC6514138; doi:10.3389/fmicb.2019.00918)
Supplement: Supplementary file 15 [file Data_Sheet_1.PDF]

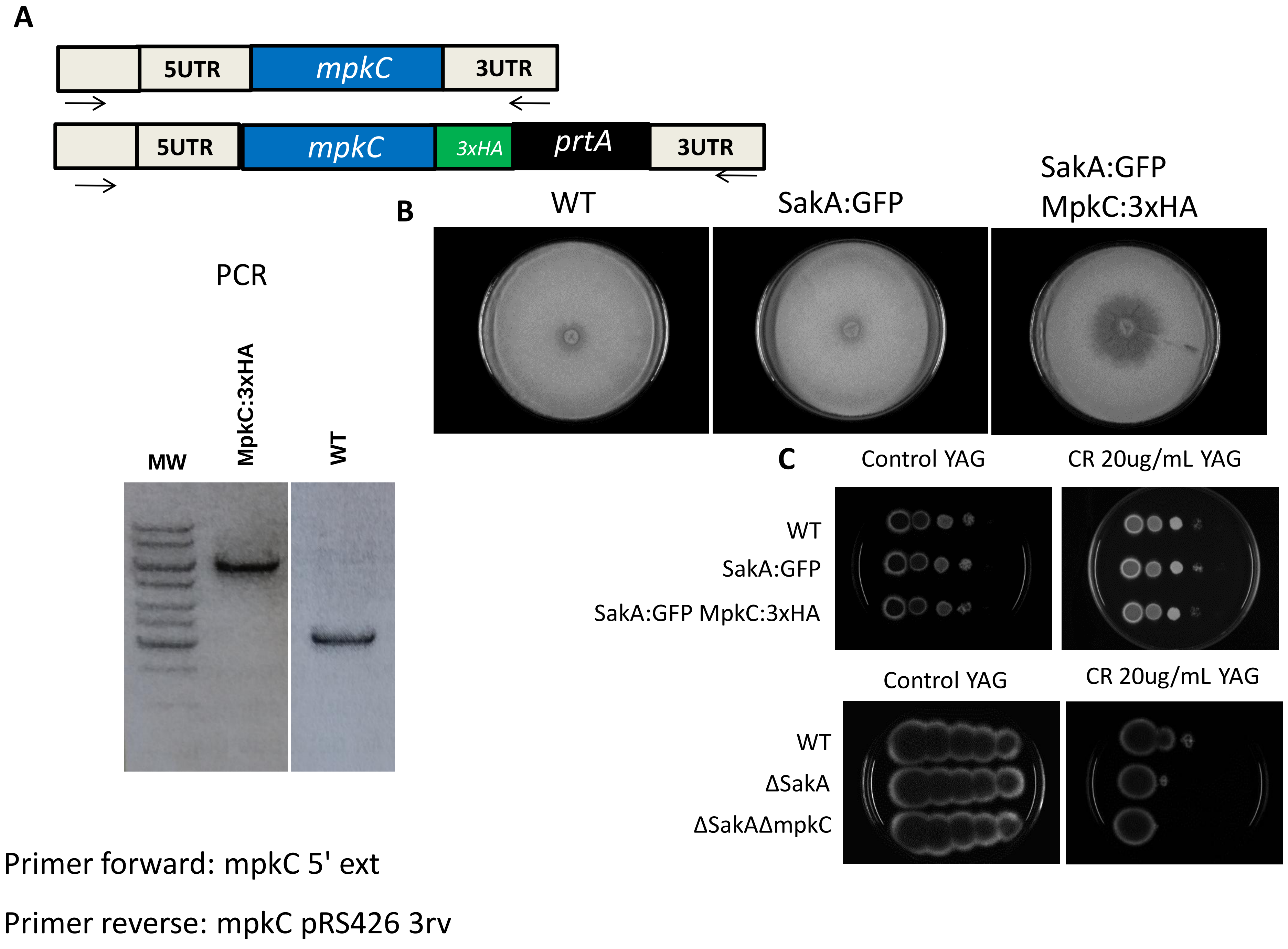

Figure S1- (A) PCR schemes to check the SakA:GFP MpkC:3xHA strains. (B) Phenotype analysis of wild-type, SakA:GFP and SakA:GFP MpkC:3xHA strains which were grown in MM plates for 4 days at 37°C. (C) Drop out experiments were performed using five µl of a tenfold dilution series starting at a concentration of 2×107 for the wild-type and mutant strains spotted on complete medium (YAG) and in presence of congo red 20 µg/mL and grown for 48 h at 37°C.
