# Supplementary material for: Aspergillus fumigatus High Osmolarity Glycerol Mitogen Activated Protein Kinases SakA and MpkC Physically Interact During Osmotic and Cell Wall Stresses
Source: Front Microbiol. 2019 May 7;10:918. doi: 10.3389/fmicb.2019.00918 (PMC6514138; doi:10.3389/fmicb.2019.00918)
Supplement: Supplementary file 17 [file Data_Sheet_3.PDF]

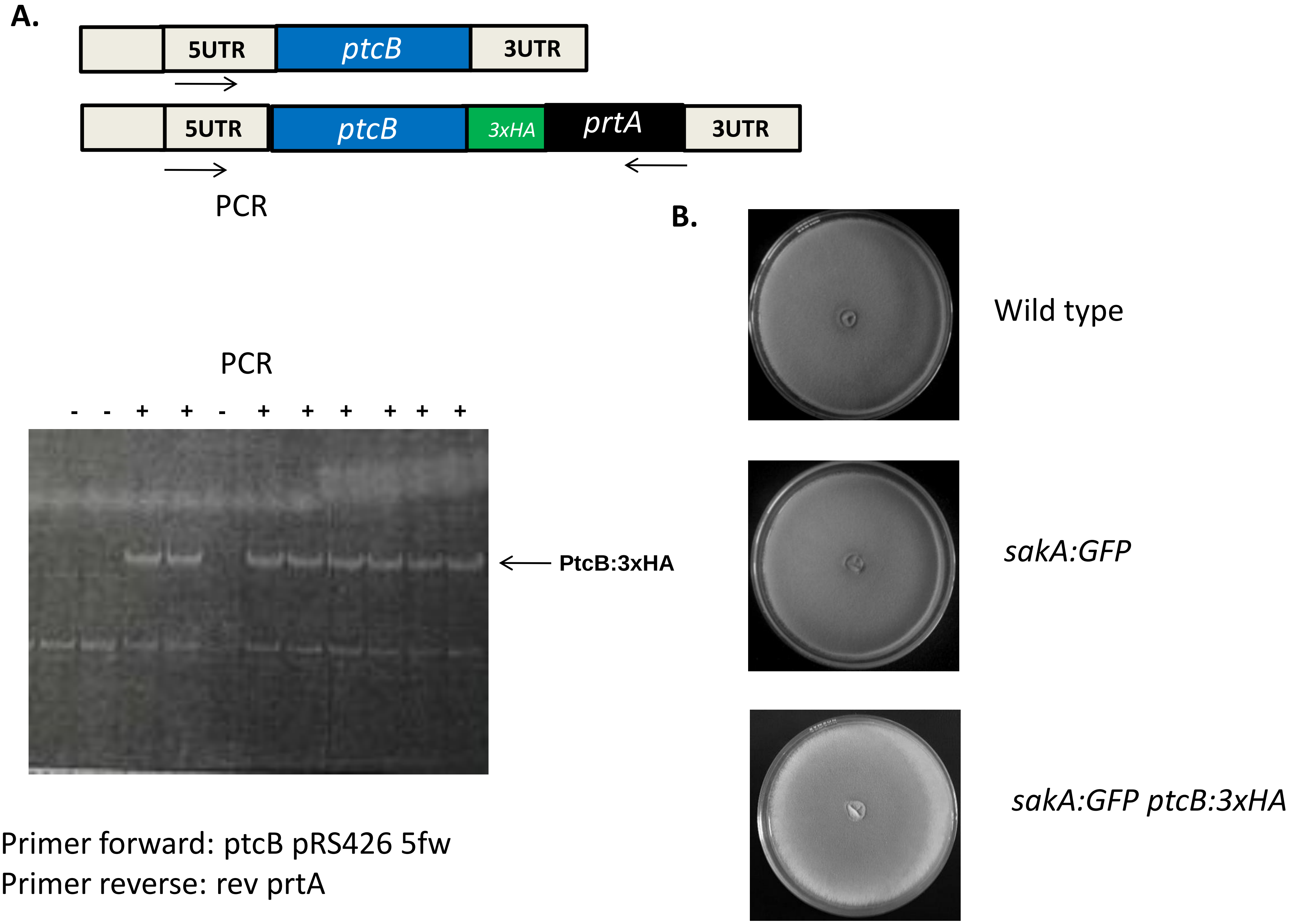

Figure S3- (A) PCR schemes to check the SakA:GFP PtcB:3xHA strains. (B) Phenotype analysis of wild-type, SakA:GFP and SakA:GFP PtcB:3xHA strains which were grown in MM plates for 4 days at 37°C.
