# Supplementary material for: Aspergillus fumigatus High Osmolarity Glycerol Mitogen Activated Protein Kinases SakA and MpkC Physically Interact During Osmotic and Cell Wall Stresses
Source: Front Microbiol. 2019 May 7;10:918. doi: 10.3389/fmicb.2019.00918 (PMC6514138; doi:10.3389/fmicb.2019.00918)
Supplement: Supplementary file 18 [file Data_Sheet_4.PDF]

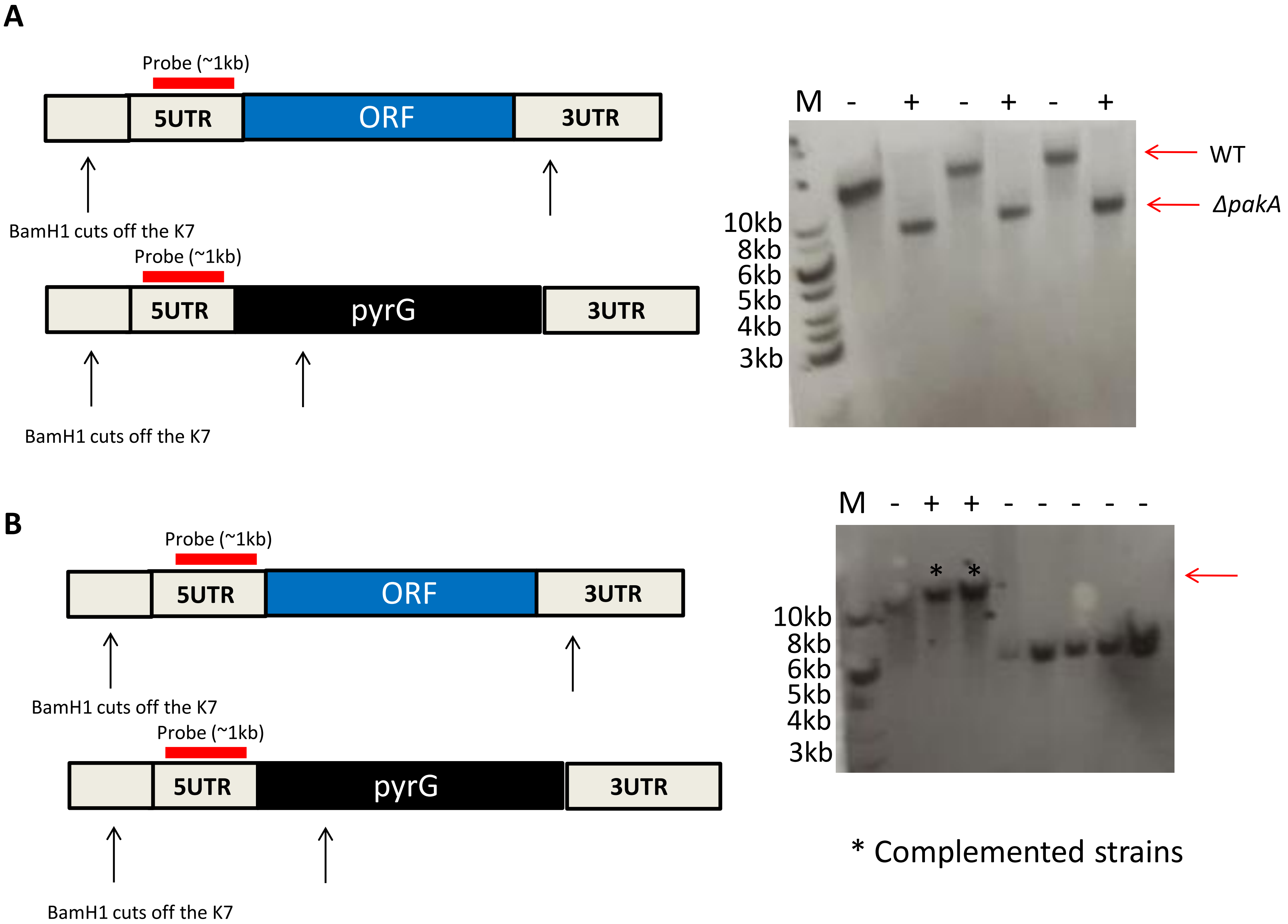

Figure S4- Deletion and complementation cassettes assembly and southern blot analysis for the *ΔpakA* null mutant (A) and complemented Δ*pakA::pakA*$^{+}$ strain (B).
